# Short-term hepatocyte function and portal hypertension outcomes of sofosbuvir/velpatasvir for decompensated hepatitis C-related cirrhosis

**DOI:** 10.1007/s00535-023-01963-2

**Published:** 2023-02-02

**Authors:** Kohei Kotani, Masaru Enomoto, Sawako Uchida-Kobayashi, Akihiro Tamori, Yoshimi Yukawa-Muto, Naoshi Odagiri, Hiroyuki Motoyama, Ritsuzo Kozuka, Etsushi Kawamura, Atsushi Hagihara, Hideki Fujii, Ken Kageyama, Akira Yamamoto, Atsushi Yoshida, Shigeaki Higashiyama, Joji Kawabe, Norifumi Kawada

**Affiliations:** 1 Department of Hepatology, Graduate School of Medicine, Osaka Metropolitan University, 1-4-3 Asahimachi, Abeno-ku, Osaka, 545-8585 Japan; 2 Department of Hepatology, Kashiwara Municipal Hospital, 1-7-9 Houzenji, Kashiwara, Osaka 582-0005 Japan; 3 Department of Diagnostic and Interventional Radiology, Graduate School of Medicine, Osaka Metropolitan University, Osaka, Japan; 4 Department of Nuclear Medicine, Graduate School of Medicine, Osaka Metropolitan University, Osaka, Japan

**Keywords:** Hepatitis C, Liver cirrhosis, Portal hypertension, Sofosbuvir, Velpatasvir

## Abstract

**Background:**

It is unclear whether hepatocyte function and/or portal hypertension improves if a sustained virologic response (SVR) is achieved with direct-acting antivirals in patients with decompensated hepatitis C-related cirrhosis.

**Methods:**

We examined the safety and efficacy of a 12-week course of sofosbuvir/velpatasvir (SOF/VEL) in 20 patients with decompensated hepatitis C-related cirrhosis. We also investigated changes in the hepatocyte receptor index (LHL15) and blood clearance index (HH15) by Tc-99 m-galactosyl human serum albumin scintigraphy, liver stiffness measurement (LSM) by transient elastography, and hepatic venous pressure gradient (HVPG) in patients who achieved an SVR at 24 weeks after treatment (SVR24).

**Results:**

One patient discontinued treatment because of rectal variceal hemorrhage, and 19 patients completed treatment. SVR24 was achieved in 17 patients (89%). Median LHL15 increased from 0.72 pre-treatment to 0.82 after SVR24 (*p* = 0.012), and median HH15 decreased from 0.82 pre-treatment to 0.76 after SVR24 (*p* = 0.010). The percentage of patients with LSM ≥ 20 kPa was 90% before treatment and remained at 90% after SVR24. However, the percentage with severe portal hypertension (defined as HVPG ≥ 12 mmHg) decreased from 92% pre-treatment to 58% after SVR24 (*p* = 0.046). Patients with a decreased HVPG from pre-treatment to after SVR24 had a smaller pre-treatment spleen volume than those with an increased HVPG (median, 252 vs. 537 mL, *p* = 0.028).

**Conclusion:**

Achieving SVR24 with SOF/VEL treatment in patients with decompensated hepatitis C-related cirrhosis can be expected to improve hepatocyte function and portal hypertension on short-term follow-up.

**Supplementary Information:**

The online version contains supplementary material available at 10.1007/s00535-023-01963-2.

## Introduction

It has become clear that a high sustained virologic response (SVR) rate can be achieved even in patients with cirrhosis owing to the recent development of direct-acting antivirals (DAAs) for hepatitis C virus (HCV). In patients with compensated cirrhosis who achieve SVR, decompensation events, such as ascites, variceal exacerbation, or hepatic encephalopathy, are suppressed by improving hepatic functional reserve [1–4]. Additionally, several reports have indicated that improvement of hepatic fibrosis, portal hypertension, and prognosis can be expected in patients with compensated cirrhosis [5–8].

In 2019, sofosbuvir/velpatasvir (SOF/VEL) treatment was approved in Japan for patients with decompensated HCV-related cirrhosis. Sofosbuvir is a nonstructural protein 5B (NS5B) polymerase inhibitor, and velpatasvir is a new-generation nonstructural protein 5A (NS5A) inhibitor. Guidelines from the American Association for the Study of Liver Diseases (AASLD), European Association for the Study of the Liver (EASL), and Japan Society of Hepatology (JSH) have recommended the use of fixed-dose combination therapy of these two DAAs for patients with decompensated cirrhosis [9–11]. In a phase 3 trial evaluating the efficacy of SOF/VEL treatment for HCV-infected patients with decompensated cirrhosis, the SVR rate was 92%, indicating that a high SVR rate can be expected, similar to that observed in patients with compensated cirrhosis [12]. When SVR is achieved in patients with decompensated cirrhosis, serum albumin levels increase, and an improved prognosis is expected [12, 13]. However, there is no consensus on whether hepatocyte function and/or portal hypertension improve when SVR is achieved with DAA treatment in patients with decompensated cirrhosis.

The recent Baveno VII workshop emphasized the importance of personalized care for portal hypertension, as the various disease stages are associated with differing outcomes, including the risk of death [14]. At Baveno VII, the definition and prevention of further decompensation in patients with decompensated cirrhosis were newly specified. Cirrhosis recompensation was also defined. This concept reflects at least partial regression of the structural and functional changes of cirrhosis after removing the cause of cirrhosis and is defined by the presence of three criteria: (1) removal of the primary etiology of cirrhosis, (2) resolution of ascites and encephalopathy and an absence of recurrent variceal hemorrhage, and (3) stable improvement of liver function tests. In patients with decompensated HCV-related cirrhosis, it is important to clarify the amount of recompensation achieved by eliminating HCV with DAA treatment.

In this study, we investigated changes in hepatocyte function and portal hypertension in patients with HCV-related decompensated cirrhosis before SOF/VEL treatment and after achieving SVR at 24 weeks after completing treatment (SVR24) in an attempt to clarify short-term outcomes. Our assessments included evaluation of hepatocyte receptor index (LHL15) and blood clearance index (HH15) by Tc-99 m-galactosyl human serum albumin (GSA) scintigraphy, liver stiffness measurement (LSM) by transient elastography (TE) (FibroScan^®^; Echosens, Paris, France), and hepatic venous pressure gradient (HVPG).

## Materials and methods

### Patients and study design

This is an observational study of patients with decompensated HCV-related cirrhosis who initiated SOF/VEL treatment at our institution. The patients underwent laboratory tests, computed tomography (CT), Tc-99 m-GSA scintigraphy, TE, and HVPG measurements within 3 months before SOF/VEL therapy. SOF/VEL (400/100 mg) fixed-dose combination treatment (EPUCLUSA^®^; Gilead Sciences, Foster City, CA, USA) was administered once daily for 12 weeks. In patients who achieved SVR24, we performed repeat laboratory tests, CT, Tc-99 m-GSA scintigraphy, TE, and HVPG measurements.

Portosystemic shunt diameter, liver volume, spleen volume, and liver-to-spleen volume ratio (L/S ratio) were measured based on pre-treatment CT data using SYNAPSE VINCENT, version 4.1, volume analyzer software (Fujifilm Medical Co., Ltd., Tokyo, Japan). HCV genotypes were identified using an HCV genotype primer kit (Institute of Immunology Co., Ltd., Tokyo, Japan), and serum HCV RNA was measured using the TaqMan HCV assay (COBAS TaqMan assay, Roche Molecular Diagnostics, Tokyo, Japan), with a lower limit of quantification of 15 IU/mL [15].

Written informed consent was obtained from all patients prior to inclusion in this study. The study was conducted in accordance with the ethical guidelines of the 1964 Declaration of Helsinki (2013 revision) and with the approval of our institution’s ethics committee (approval no. 4292).

### Measurement of Tc-99 m-GSA scintigraphy

With the patients in the supine position, 185 MBq of Tc-99 m-GSA (Nihon Medi-Physics Co. Ltd., Tokyo, Japan) was injected through a cubital vein, and planar images were dynamically acquired in frames of 60 s, beginning immediately after injection and continuing for 20 min. A scintillation camera (Brightview X system; Philips Medical Systems Inc., Cleveland, OH, USA) was used to generate time-activity curves. This camera had a low-energy, high-resolution collimator and was interfaced with an extended brilliance workspace-nuclear medicine workstation. The images were constructed and analyzed using asialo analysis of the interface definition language sample application software, version 2.0.1, supplied by the Hitachi Medical Corporation (Tokyo, Japan). Time–activity curves were drawn with regions of interest set in the liver and heart on the 20-min integrated images. According to the previously reported method [16, 17], LHL15 and HH15 were determined as follows: LHL15, liver uptake of radiotracer at 15 min/(liver plus heart uptake of radiotracer at 15 min); and HH15, heart pooling of radiotracer at 15 min/heart pooling of radiotracer at 3 min.

### Measurement of HVPG

A 5-French cobra-shaped Selecon MP balloon catheter (Terumo Clinical Supply Co., Ltd., Gifu, Japan) was inserted via the jugular vein or antecubital vein into the hepatic vein, and the mean free hepatic venous pressure (FHVP) was measured. The balloon was then inflated, and the average wedged hepatic venous pressure (WHVP) was determined. Both FHVP and WHVP were measured three times, and the mean values were calculated. HVPG was calculated as mean WHVP minus mean FHVP [18]. HVPG measurements were performed safely in all patients.

### Statistical analysis

JMP 11.0.0 statistical software (SAS Institute Inc., Cary, NC, USA) was used for statistical analyses. Continuous variables were expressed as median (interquartile range). The Mann–Whitney *U* test was used to compare continuous variables between 2 groups, while Fisher’s exact test or Chi-squared test was used to compare categorical variables. Correlations between continuous variables were analyzed using Spearman’s rank correlation coefficient (*ρ*). The Wilcoxon signed-rank test was used to compare continuous variables before and after SOF/VEL treatment, while the Bowker test was used to compare categorical variables before and after SOF/VEL treatment. *p* values < 0.05 were considered statistically significant.

## Results

### Baseline characteristics

**Fig. 1:**
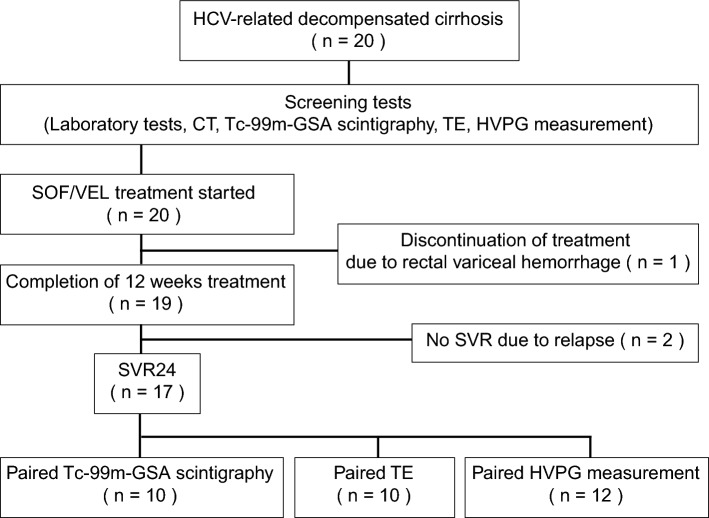
Study flowchart. *HCV* hepatitis C virus, *CT* computed tomography, *Tc-99 m-GSA* technetium-99 m-galactosyl human serum albumin, *TE* transient elastography, *HVPG* hepatic venous pressure gradient, *SOF* sofosbuvir, *VEL* velpatasvir, *SVR24* sustained virologic response at 24 weeks after treatment

Between January 2019 and March 2021, 20 patients with decompensated HCV-related cirrhosis were enrolled and started SOF/VEL treatment. A total of 19 patients completed treatment and were observed for at least 24 weeks after the end of treatment. Because of the invasiveness of the procedures, radiation exposure, and/or retention of massive ascites, paired (before and after treatment) assessments were not performed in all patients who achieved SVR, but they were available as follows: scintigraphy, 10 patients; TE, 10 patients; and HVPG measurements, 12 patients (Fig. 1).

The clinical characteristics of all patients are summarized in Table 1. The median age was 63 years, and there were 13 males and 7 females. A total of 16 patients (80%) had esophageal varices, and 10 patients (50%) had a history of hepatocellular carcinoma (HCC). The HCV genotype was 1b in 15 patients (75%), 2a in 2 patients (10%), and 2b in 3 patients (15%). The Child–Pugh–Turcotte (CPT) category was grade B in 17 patients (85%), grade C in 3 patients (15%), and grade A in 0 patients. The median LHL15 (reflecting hepatocyte function) was 0.69, median LSM (reflecting liver stiffness) was 37 kPa, and median HVPG (reflecting portal pressure) was 17 mmHg.

**Table 1:** Baseline characteristics of all patients (*n* = 20)

Characteristic	Value
Sex (male/female)	13/7
Age (y)	63 (60–72)
Esophageal varices (yes/no)	16/4
History of HCC (yes/no)	10/10
HCV RNA (log_10_ IU/mL)	6.0 (5.7–6.2)
HCV genotype (1b/2a/2b)	15/2/3
CPT score	8 (7–9)
CPT grade (A/B/C)	0/17/3
ALBI score	− 1.39 (− 1.11 to − 1.78)
ALBI grade (1/2/3)	0/10/10
LHL15	0.69 (0.60–0.74)
HH15	0.83 (0.79–0.85)
LSM (kPa)	37 (27–45)
HVPG (mmHg)	17 (13–19)

Data are shown as numbers or median (interquartile range)

*HCC* hepatocellular carcinoma, *HCV* hepatitis C virus, *CPT* Child–Pugh–Turcotte, *ALBI* albumin–bilirubin, *LHL15* hepatocyte receptor index, *HH15* blood clearance index, *LSM* liver stiffness measurement, *HVPG* hepatic venous pressure gradient

### Safety and efficacy of SOF/VEL treatment

During SOF/VEL treatment, the following adverse events were observed: ascites or edema in seven patients (35%), elevated serum ammonia level in three patients (15%), exacerbation or rupture of esophago-gastrointestinal varices in three patients (15%), portal thrombosis in one patient (5%), and leg cramps in one patient (5%). However, all of these events were determined to be caused by decompensated cirrhosis, not by SOF/VEL treatment. One patient discontinued treatment because of rectal variceal hemorrhage, and 19 patients completed the 12-week course of treatment. SVR24 was achieved in 17 patients (89%) (Fig. 1).

### Changes in laboratory tests and endoscopy results

Serum aspartate aminotransferase (AST) (*p* < 0.001), alanine aminotransferase (ALT) (*p* < 0.001), albumin (*p* = 0.001), prothrombin time–international normalized ratio (*p* = 0.018), CPT score (*p* = 0.049), albumin–bilirubin (ALBI) score (*p* = 0.002), type 4 collagen 7S domain (*p* = 0.006), and mac-2 binding protein glycosylation isomer (M2BPGi) (*p* < 0.001) improved at SVR24, compared with pre-treatment. No patient had CPT grade A before SOF/VEL treatment, but ten patients (59%) improved to CPT grade A at SVR24 (*p* = 0.019) (Table 2). Of the 14 patients taking diuretics pre-treatment, 8 (57%) were able to reduce the dose of diuretics after treatment because of improved ascites or edema. In the 15 patients with esophageal varices pre-treatment, follow-up esophagogastroduodenoscopy revealed improved varices in 5 (33%), no change in varices in 5 (33%), and worsened variceal morphology in 3 (20%); 2 patients (13%) did not undergo esophagogastroduodenoscopy after viral elimination.

**Table 2:** Changes in laboratory tests from before SOF/VEL treatment to after SVR24 (*n* = 17)

Test	Before treatment	SVR24	*p*
AST (U/L)	47 (38–69)	28 (25–33)	< 0.001
ALT (U/L)	34 (18–46)	14 (11–19)	< 0.001
Albumin (g/dL)	2.8 (2.3–3.1)	3.4 (2.7–4.0)	0.001
Total bilirubin (mg/dL)	1.5 (1.0–2.2)	1.3 (0.8–2.0)	0.316
PT–INR	1.21 (1.14–1.30)	1.15 (1.09–1.22)	0.018
Esophageal varices (yes/no/NA)	15/2/0	13/1/3	0.392
NH_3_ (μg/dL)	75 (51–109)	74 (53–89)	0.424
Ascites (no/mild/moderate)	9/4/4	10/5/2	0.572
Platelets (× 10^9^/L)	68 (57–108)	79 (40–112)	0.669
CPT score	8 (7–9)	6 (6–9)	0.049
CPT grade (A/B/C)	0/14/3	10/6/1	0.019
ALBI score	− 1.29 (− 1.04 to − 1.82)	− 2.03 (− 1.35 to − 2.45)	0.002
ALBI grade (1/2/3)	0/8/9	3/10/4	0.123
MELD score	10 (9–13)	9 (8–12)	0.211
Type 4 collagen 7S (ng/mL)	14.4 (8.8–15.7)	9.2 (7.8–12.5)	0.006
M2BPGi (C.O.I)	14.0 (10.2–16.2)	7.7 (4.2–12.1)	< 0.001
FIB-4 index	7.06 (5.85–11.73)	6.27 (4.06–10.30)	0.057

Data are shown as number and median (interquartile range) and were compared using the Bowker test or Wilcoxon signed-rank test or test, respectively

*SOF* sofosbuvir, *VEL* velpatasvir, *SVR* sustained virologic response, *AST* aspartate aminotransferase, *ALT* alanine aminotransferase, *PT* prothrombin time, *INR* international normalized ratio, *NA* not assessed, *NH*_*3*_ ammonia, *CPT* Child–Pugh–Turcotte, *ALBI* albumin–bilirubin, *MELD* model for end-stage liver disease, *M2BPGi* mac-2 binding protein glycosylation isomer

### Changes in Tc-99 m-GSA scintigraphy, LSM, and HVPG

**Fig. 2:**
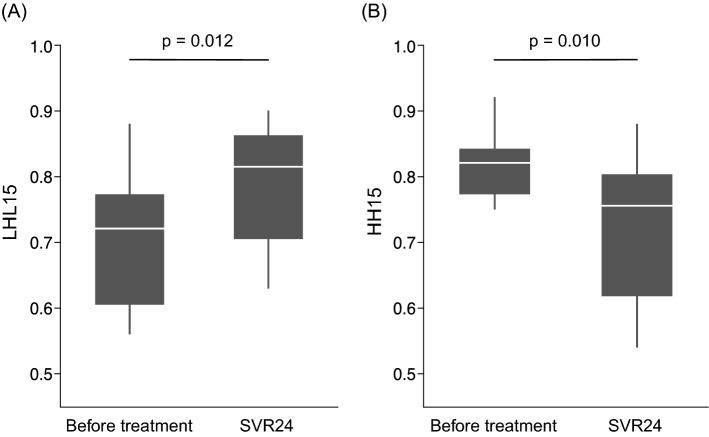
Changes in Tc-99 m-galactosyl human serum albumin scintigraphy data before sofosbuvir/velpatasvir treatment and after sustained virologic response at 24 weeks post-treatment (SVR24). Hepatocyte receptor index (LHL15) increased from 0.72 (0.61–0.77) to 0.82 (0.71–0.86) (*p* = 0.012) (**a**), and blood clearance index (HH15) decreased from 0.82 (0.77–0.84) to 0.76 (0.62–0.80) (*p* = 0.010) (**b**), after SVR24 compared with before treatment

**Fig. 3:**
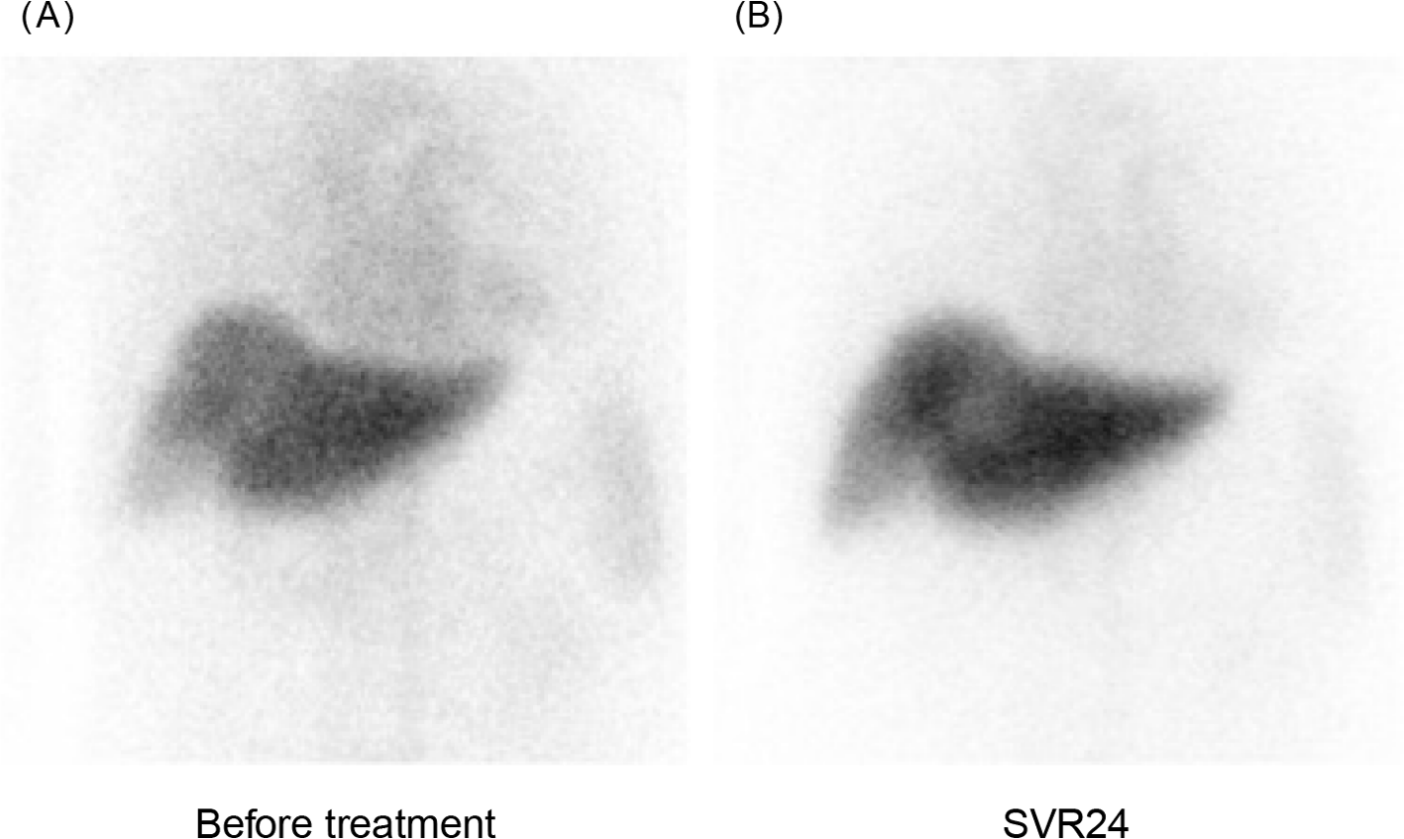
Tc-99 m-galactosyl human serum albumin scintigraphic images obtained from a 65-year-old man with genotype 2b infection before sofosbuvir/velpatasvir treatment and after sustained virologic response at 24 weeks post-treatment (SVR24). Before treatment, the planar image shows weak accumulation of radiotracer in the liver and enhanced pooling in the heart; hepatocyte receptor index (LHL15) and blood clearance index (HH15) were 0.70 and 0.78, respectively (**a**). After SVR24, liver accumulation of radiotracer increased and cardiac pooling decreased; LHL15 and HH15 improved to 0.85 and 0.63, respectively (**b**). The patient’s hepatic functional reserve improved from Child–Pugh-Turcotte grade B to grade A after SVR24

Both LHL15 and HH15 improved in nine patients (90%) and did not improve in one patient (10%) after SVR24, compared with pre-treatment. Median LHL15 increased from 0.72 pre-treatment to 0.82 after SVR24 (*p* = 0.012), and median HH15 decreased from 0.82 before treatment to 0.76 after SVR24 (*p* = 0.010) (Fig. 2). The median percentage increase in LHL15 was 14%, and the median percentage decrease in HH15 was 9%. The median percentage increase in LHL15 did not differ between patients with CPT grade B and grade C disease (*p* = 0.602) or between patients with and without a history of HCC (*p* = 0.347). Similarly, the median percentage decrease in HH15 did not differ between patients with CPT grade B and grade C disease (*p* = 0.794) or between patients with and without a history of HCC (*p* = 0.917). A representative case in which hepatocyte function improved is shown in Fig. 3, and a patient with no improvement in hepatocyte function is described in Supplementary Fig. 1.

LSM decreased in 6 patients (60%) and increased in 4 patients (40%) after SVR24, compared with pre-treatment. Baseline characteristics of the decreased LSM group and the increased LSM group are shown in Supplementary Table 1. Median LSM decreased from 35 kPa pre-treatment to 27 kPa after SVR24, but this decrease was not significant (*p* = 0.719) (Supplementary Fig. 2). The median percentage decrease in LSM was 12%. The percentage of patients with an LSM ≥ 20 kPa (defined as the cut-off value for risk of having endoscopic signs of portal hypertension according to the Baveno VI criteria [19]) was 90% before treatment and remained at 90% after SVR24 (Supplementary Fig. 3).

**Fig. 4:**
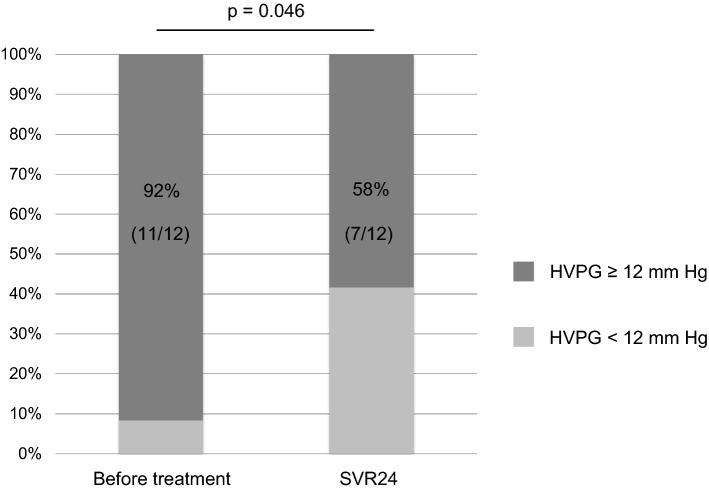
Changes in percentages of patients with severe portal hypertension, defined as hepatic venous pressure gradient (HVPG) ≥ 12 mmHg, before sofosbuvir/velpatasvir treatment and after sustained virologic response at 24 weeks post-treatment (SVR24). The percentage was 92% (11/12) before treatment and significantly decreased to 58% (7/12) after SVR24 (*p* = 0.046)

HVPG decreased in eight patients (67%) after SVR24, compared with pre-treatment. The decrease was ≥ 10% in all eight patients and ≥ 20% in five patients (42%). However, HVPG increased in four patients (33%) after SVR24, compared with pre-treatment. Median HVPG decreased from 18 mmHg pre-treatment to 15 mmHg after SVR24 (*p* = 0.119) (Supplementary Fig. 4), and the median percentage decrease in HVPG was 15%. The median percentage decrease in HVPG did not differ between patients with CPT grade B and grade C disease (*p* = 0.667) or between patients with and without a history of HCC (*p* = 0.223). The percentage of patients with clinically significant portal hypertension (CSPH), defined as HVPG ≥ 10 mmHg, was 92% before treatment and decreased to 75% after SVR24 (*p* = 0.157) (Supplementary Fig. 5). However, these results were not statistically significant. Conversely, the percentage of patients with severe portal hypertension, defined as HVPG ≥ 12 mmHg, was 92% before treatment and decreased significantly to 58% after SVR24 (*p* = 0.046) (Fig. 4).

### Relationship between CT findings and changes in liver function and HVPG

We also assessed the association between pre-treatment CT findings and post-treatment changes in liver function and HVPG. The median portosystemic shunt diameter was 7 mm at baseline. There was no difference in portosystemic shunt diameter between the improved CPT score group and the unchanged or worsened CPT score group (median, 8 vs. 6 mm, *p* = 0.337). There was also no correlation between shunt diameter and the degree of increase in LHL15 (*ρ* = 0.328, *p* = 0.355) or decrease in HH15 (*ρ* = 0.065, *p* = 0.859). Similarly, there was no correlation between shunt diameter and pre-treatment HVPG (*ρ* = 0.066, *p* = 0.800) or the degree of decrease in HVPG (*ρ* = 0.155, *p* = 0.630).

The decreased HVPG group had a smaller spleen volume (median, 252 vs. 537 mL, *p* = 0.028) and larger L/S ratio (median, 4.5 vs. 1.8, *p* = 0.028) at baseline than the increased HVPG group. However, there were no differences in pre-treatment CPT score, ALBI score, LHL15, HH15, LSM, and HVPG between the decreased and increased HVPG groups (Table 3).

**Table 3:** Baseline characteristics of the decreased HVPG group and increased HVPG group (*n* = 12)

Characteristic	Decreased HVPG (*n* = 8)	Increased HVPG (*n* = 4)	*p*
CPT score	8 (7–9)	7 (7–9)	0.413
ALBI score	− 1.55 (− 1.19 to − 2.01)	− 1.25 (− 0.77 to − 1.60)	0.235
MELD score	9 (8–12)	11 (10–13)	0.120
Type 4 collagen 7S (ng/mL)	11.7 (8.5–15.8)	15.0 (14.4–16.0)	0.235
M2BPGi (C.O.I)	13.1 (5.8–18.5)	14.0 (9.6–17.4)	0.734
FIB-4 index	6.60 (4.79–10.81)	10.89 (10.07–13.55)	0.126
PSS diameter (mm)	8 (6–9)	7 (5–13)	0.798
Liver volume (mL)	1044 (864–1193)	997 (858–1247)	0.831
Spleen volume (mL)	252 (183–343)	537 (403–1037)	0.028
L/S ratio	4.5 (2.7–6.0)	1.8 (1.2–2.4)	0.028
LHL 15	0.72 (0.66–0.80)	0.68 (0.60–0.77)	0.748
HH15	0.81 (0.73–0.85)	0.83 (0.77–0.85)	0.669
LSM (kPa)	30 (23–39)	36 (33–43)	0.364
HVPG (mmHg)	18 (14–20)	18 (15–21)	0.798

Data are shown as median (interquartile range) and were compared using the Mann–Whitney *U* test

*HVPG* hepatic venous pressure gradient, *CPT* Child–Pugh–Turcotte, *ALBI* albumin–bilirubin, *MELD* model for end-stage liver disease, *M2BPGi* mac-2 binding protein glycosylation isomer, *PSS* portosystemic shunt, *L/S ratio* liver-to-spleen volume ratio, *LHL15* hepatocyte receptor index, *HH15* blood clearance index, *LSM* liver stiffness measurement

Median spleen size changed from 365 mL before treatment to 332 mL after SVR24 (*p* = 0.132), and median L/S ratio changed from 2.5 at pre-treatment to 2.8 after SVR24 (*p* = 0.244), although these changes were not statistically significant. There was no correlation between change in spleen size and change in HVPG from before to after treatment (*ρ* = –0.424, *p* = 0.194).

## Discussion

Management of decompensated cirrhosis focuses on preventing cirrhosis progression. The most important strategy involves suppression of the etiologic factors that have caused liver inflammation and subsequent fibrosis [20]. In patients with HCV-related decompensated cirrhosis, SVR is required to reduce liver inflammation and improve liver function. Several studies have been previously published regarding the efficacy and outcomes of SOF/VEL treatment in patients with decompensated cirrhosis [21–23]. While high SVR rates have been reported with DAA treatment for decompensated cirrhosis, the effects of SVR on hepatocyte function and portal hypertension have not been heretofore studied in detail [12, 13]. This is the first study reporting hepatocyte function before and after DAA treatment using Tc-99 m-GSA scintigraphy in patients with HCV-related cirrhosis. Furthermore, since we targeted only patients with decompensated cirrhosis (CPT grades B or C), this study evaluated changes in LSM and HVPG before and after treatment in patients with more advanced liver disease than those included in previous studies [24–28].

Regarding the efficacy of SOF/VEL treatment for patients with decompensated HCV-related cirrhosis, SVR12 rates were 83% to 92% in a phase 3 study [12, 29] and 90% to 93% in real-world data [13, 30]. In our study, the SVR24 rate was 89%, which was comparable to these previous results. Prior studies reported that adverse events associated with SOF/VEL treatment were nonspecific, such as fatigue, headache, nausea, and appetite loss [29, 30]. In this study, ascites, edema, elevated serum ammonia level, and exacerbation of esophago-gastrointestinal varices were observed during SOF/VEL treatment, but these adverse events are often experienced during cirrhosis decompensation itself. They can be managed by implementing usual cirrhosis treatment and do not require SOF/VEL-specific treatment. Our results, therefore, suggest that it is safe to administer SOF/VEL, even in patients with decompensated cirrhosis.

It is well known that antiviral treatment improves liver function indices, such as serum albumin, CPT score, and ALBI score, but it is necessary to verify whether this is due to improvement of hepatocyte function or the effects of other factors, such as improved nutrition, biliary stenosis, or cholestasis. Tc-99 m-GSA scintigraphy results directly reflect hepatocyte function and are less likely to be affected by extrahepatic factors since GSA specifically binds to asialoglycoprotein receptors (ASGPRs) on the surface of each hepatocyte [16, 17]. Kira et al. found that patients with chronic hepatitis C treated with interferon-α exhibited improved Tc-99 m-GSA scintigraphy data after SVR [31]. Similarly, Ishii et al. described a patient with chronic hepatitis C whose Tc-99 m-GSA scintigraphy results improved with low-dose long-term interferon-α treatment [32]. No previously published study used Tc-99 m-GSA scintigraphy to evaluate hepatocyte function in patients with hepatitis C treated with DAAs. We found that SOF/VEL improved both LHL15 and HH15, indicating that this combination treatment improves hepatocyte function in the short term, even in patients with decompensated cirrhosis. ASGPRs may play a role in this improved hepatocyte function. ASGPRs are expressed in a polar manner on the sinusoidal and basolateral surfaces of the hepatocyte plasma membrane [33, 34]. Expression of ASGPRs is altered by various cytokines, and ASGPR expression on the sinusoidal surface is decreased in cirrhosis [35, 36]. Suppression of hepatitis by DAA treatment may reduce inflammatory cytokines and preserve ASGPR function, thereby restoring the differentiation and proliferation potential of residual hepatocytes and improving the ability to synthesize albumin, prothrombin, and other substances. Decompensated cirrhosis often develops in patients with esophagogastric varices and/or HCC, and effective treatment for these patients can be indicated if hepatic function is preserved. Although longer observation periods are required to assess the impact of improved hepatocyte function on longer-term outcomes of patients with HCV-related decompensated cirrhosis, SOF/VEL treatment should be considered if esophagogastric varices and/or HCC are controlled.

We found that AST, ALT, albumin, CPT score, and ALBI score improved after SVR24, as in previous reports [12, 13, 30], and that liver fibrosis markers, such as type 4 collagen 7S and M2BPGi, also improved. Serum markers for liver fibrosis have been previously reported to improve with achieving SVR in chronic hepatitis and compensated cirrhosis [37–39]. However, fibrosis markers elevate because of not only liver fibrosis but also liver inflammation [39–41]. Poordad et al. reported that 71% of patients with cirrhosis had a histologically proven downgraded METAVIR score at 52 weeks after DAA treatment [42]. In contrast, we previously reported that decreased serum type 4 collagen 7S after SVR was related to an improved liver inflammatory response, while no significant regression of histologic fibrosis stage was noted at 41 ± 20 weeks after DAA treatment in patients with chronic hepatitis C [43]. Our current results also showed that LSM did not improve at SVR24. Taken together, these results suggest that regression of liver fibrosis is limited or absent in decompensated cirrhosis in the short term, although we did not examine liver histology to confirm this.

Previous studies have shown that HVPG decreases when SVR is achieved in patients with HCV-related cirrhosis [24–28]. Mandorfer et al. analyzed 60 patients with cirrhosis treated with DAAs and found that HVPG decreased significantly from 13.1 mmHg pre-treatment to 10.4 mmHg after SVR, but the amount of HVPG decrease was small in patients with CPT stage B disease [26]. In their study of 226 patients with CSPH, Lens et al. found that the median HVPG decreased from 15 mmHg at baseline to 13 mmHg at SVR24, but CSPH persisted in the majority of patients at this time [27], as well as in a follow-up study conducted 96 weeks after treatment [28]. In those studies, the proportion of patients with CPT grade A cirrhosis was high. In contrast, all patients in our study had CPT grade B or C decompensated cirrhosis, so baseline HVPG was high (17 mmHg). CSPH represents the risk of clinical decompensation [44], and HVPG ≥ 12 mmHg is the cut-off value associated with a high risk of variceal bleeding and other complications of portal hypertension [45, 46]. In addition, a reduction in HVPG to ≤ 12 mmHg or by ≥ 20% from baseline was previously shown to be protective against the development of variceal bleeding and associated with a decreased incidence of decompensation [47, 48]. In our study, 42% of patients exhibited a decrease in HVPG by ≥ 20%, and the percentage of patients with severe portal hypertension (HVPG ≥ 12 mmHg) decreased significantly from 92% before treatment to 58% after SVR. However, 75% of our study cohort of patients with CPT grade B or C cirrhosis continued to have CSPH after SVR24, which was similar to the results reported by Lens et al. whose study also included CPT grade A patients [27]. Thus, our results indicate that careful follow-up is required to monitor for the development of liver-related complications, regardless of whether SVR is achieved.

In patients with compensated cirrhosis, Tsuji et al. found that larger-diameter portosystemic shunts were associated with the development of portal hypertension-related events and impaired improvement of the liver function after DAA treatment [49]. Similarly, in 72 patients with decompensated cirrhosis treated with SOF/VEL, Takaoka et al. reported that a large portosystemic shunt (diameter ≥ 6 mm) was associated with less improvement in CPT scores [50]. In the current study, we identified no association between portosystemic shunt diameter and improvement in CPT scores, Tc-99 m-GSA scintigraphy results, or HVPG. Nevertheless, we did find that patients with a decreased HVPG after SVR24 had a smaller pre-treatment spleen volume. When the spleen is large, splenic arterial blood flow increases, resulting in increased splenic venous flow and worsened portal hypertension [51]. However, we found no association between a decreased HVPG and the pre-treatment HVPG value. One explanation may be that splenic venous flow secondary to splenomegaly does not decrease at only 24 weeks after completing treatment, so a reduction in HVPG would not be observed. Another possible explanation is that, like vascular endothelial cells, the spleen produces endothelin-1, which activates hepatic stellate cells and generates constriction of hepatic sinusoids, resulting in increased intrahepatic vascular resistance [52]. Recovery of hepatocyte function with SOF/VEL treatment may improve the hepatic microcirculatory environment, but HVPG may not decrease in patients with splenomegaly possibly because of high amounts of endothelin-1 produced by the enlarged spleen.

This study has several limitations. First, it was conducted with an observational design at a single center, so the data may be heterogeneous or insufficient. Second, the statistical power of this study may be low, and selection bias cannot be excluded because the number of patients enrolled was small, and not all patients underwent paired (pre- and post-treatment) tests. Furthermore, multivariate analyses could not be performed because of the small number of patients; therefore, it is difficult to assess the role of potential confounding factors. Third, the observation period of this study was short because data were assessed at SVR24, so the effects of SOF/VEL treatment on longer-term prognosis with regard to liver-related complications and survival are unclear. Further validation studies with a larger cohort are required to resolve these concerns in the future.

In conclusion, a 12-week course of SOF/VEL treatment for decompensated cirrhosis can eliminate HCV at a high SVR, although patients must be closely monitored for the development of complications of cirrhosis. Our results also suggest that SOF/VEL treatment in patients with decompensated HCV-related cirrhosis may restore hepatocyte function and reduce the proportion of patients with severe portal hypertension. However, the effects of SOF/VEL treatment on prognosis in terms of liver-related complications and survival requires further studies with longer-term follow-up.

## Supplementary Information

Below is the link to the electronic supplementary material.

Supplementary file1 A 72-year-old man with hepatitis C genotype 1b infection whose hepatocyte function did not improve. His pre-treatment data were as follows: Child-Pugh-Turcotte (CPT) score, 7; hepatocyte receptor index (LHL15), 0.78; blood clearance index (HH15), 0.75; liver stiffness measurement (LSM), 43 kPa; and hepatic venous pressure gradient (HVPG), 17 mmHg. Before treatment, the planar image of Tc-99m-galactosyl human serum albumin (GSA) scintigraphy showed weak accumulation of radiotracer in the liver and enhanced pooling in the heart (a). After sustained virologic response at 24 weeks post-treatment (SVR24), all of the abovementioned values were worse: CPT score, 9; LHL15, 0.73; HH15, 0.79; LSM, 75 kPa; and HVPG, 22 mmHg. Accumulation of radiotracer in the liver remained weak, and pooling of tracer in the heart did not decrease (b). CT performed after SVR24 showed recurrent hepatocellular carcinoma lesions in the right lobe, which were enhanced in the arterial phase and washed out in the late phase (arrow) (c) (PPTX 425 KB)

Supplementary file2 Liver stiffness measurement (LSM) was 35 (27–39) kPa before sofosbuvir/velpatasvir treatment and decreased to 27 (21–44) kPa after sustained virologic response at 24 weeks post-treatment (SVR24). This decrease was not statistically significant (p=0.719) (PPTX 73 KB)

Supplementary file3 Percentage of patients with a liver stiffness measurement (LSM) ≥ 20 kPa was 90% (9/10) before sofosbuvir/velpatasvir treatment and remained at 90% (9/10) after sustained virologic response at 24 weeks post-treatment (SVR24) (p=1.000) (PPTX 60 KB)

Supplementary file4 Hepatic venous pressure gradient (HVPG) was 18 (15–20) mmHg before sofosbuvir/velpatasvir treatment and decreased to 15 (9–22) mmHg after sustained virologic response at 24 weeks post-treatment (SVR24). However, this decrease was not statistically significant (p=0.119) (PPTX 76 KB)

Supplementary file5 Percentage of patients with clinically significant portal hypertension (CSPH), defined as a hepatic venous pressure gradient (HVPG) ≥ 10 mmHg, was 92% (11/12) before sofosbuvir/velpatasvir treatment and decreased to 75% (9/12) after sustained virologic response at 24 weeks post-treatment (SVR24). However, the decrease was not statistically significant (p=0.157) (PPTX 60 KB)

Supplementary file6 (DOCX 27 KB)
