# Supplementary figures and images for: Short-term hepatocyte function and portal hypertension outcomes of sofosbuvir/velpatasvir for decompensated hepatitis C-related cirrhosis
Source: J Gastroenterol. 2023 Feb 2;58(4):394–404. doi: 10.1007/s00535-023-01963-2 (PMC10049944; doi:10.1007/s00535-023-01963-2)

## Slide 1
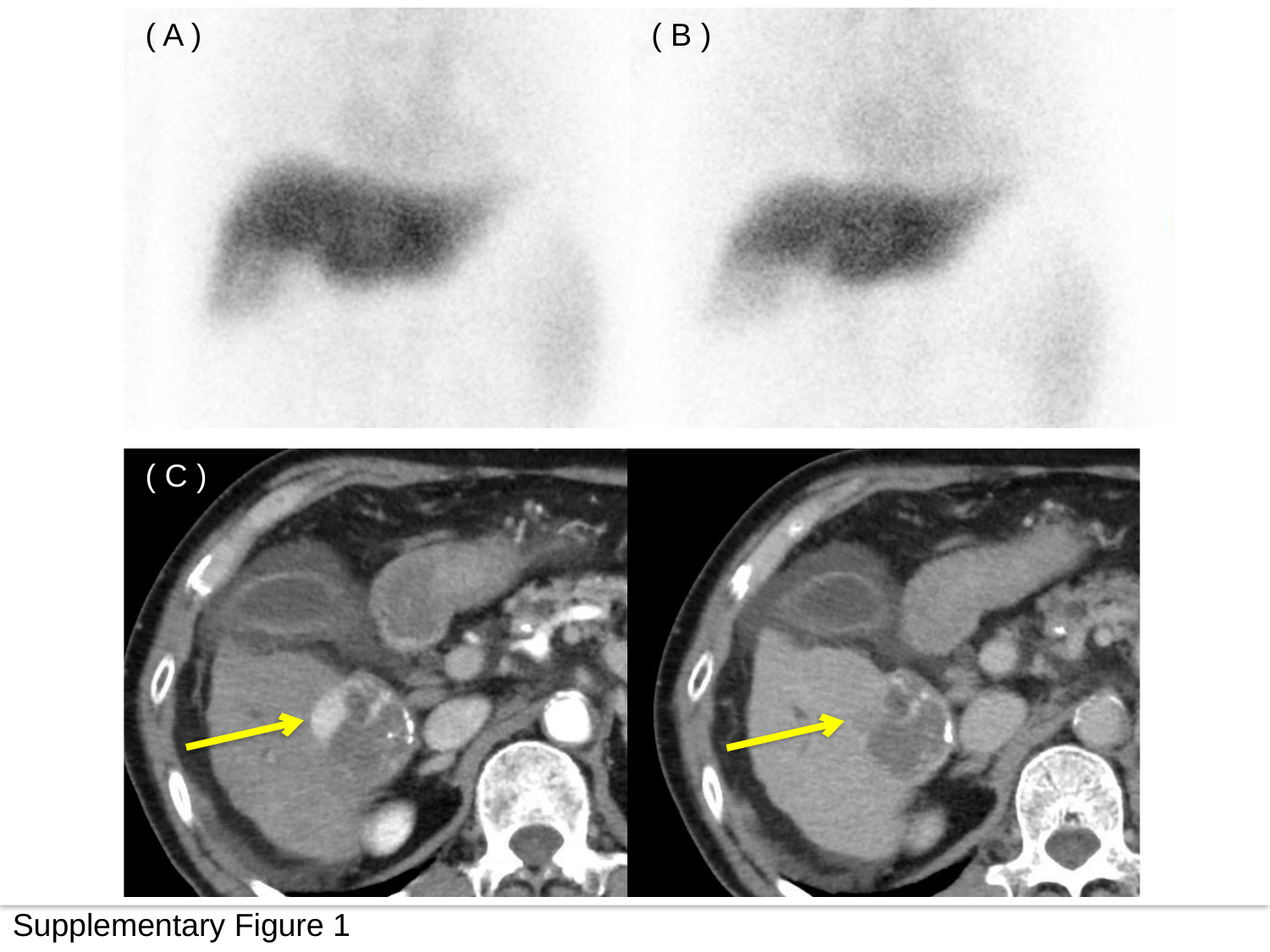

( A )
 ( B )
 ( C )
Supplementary Figure 1

Supplement: Supplementary file 1 — Supplementary file1 A 72-year-old man with hepatitis C genotype 1b infection whose hepatocyte function did not improve. His pre-treatment data were as follows: Child-Pugh-Turcotte (CPT) score, 7; hepatocyte receptor index (LHL15), 0.78; blood clearance index (HH15), 0.75; liver stiffness measurement (LSM), 43 kPa; and hepatic venous pressure gradient (HVPG), 17 mmHg. Before treatment, the planar image of Tc-99m-galactosyl human serum albumin (GSA) scintigraphy showed weak accumulation of radiotracer in the liver and enhanced pooling in the heart (a). After sustained virologic response at 24 weeks post-treatment (SVR24), all of the abovementioned values were worse: CPT score, 9; LHL15, 0.73; HH15, 0.79; LSM, 75 kPa; and HVPG, 22 mmHg. Accumulation of radiotracer in the liver remained weak, and pooling of tracer in the heart did not decrease (b). CT performed after SVR24 showed recurrent hepatocellular carcinoma lesions in the right lobe, which were enhanced in the arterial phase and washed out in the late phase (arrow) (c) (PPTX 425 KB) [file 535_2023_1963_MOESM1_ESM.pptx]

## Slide 1
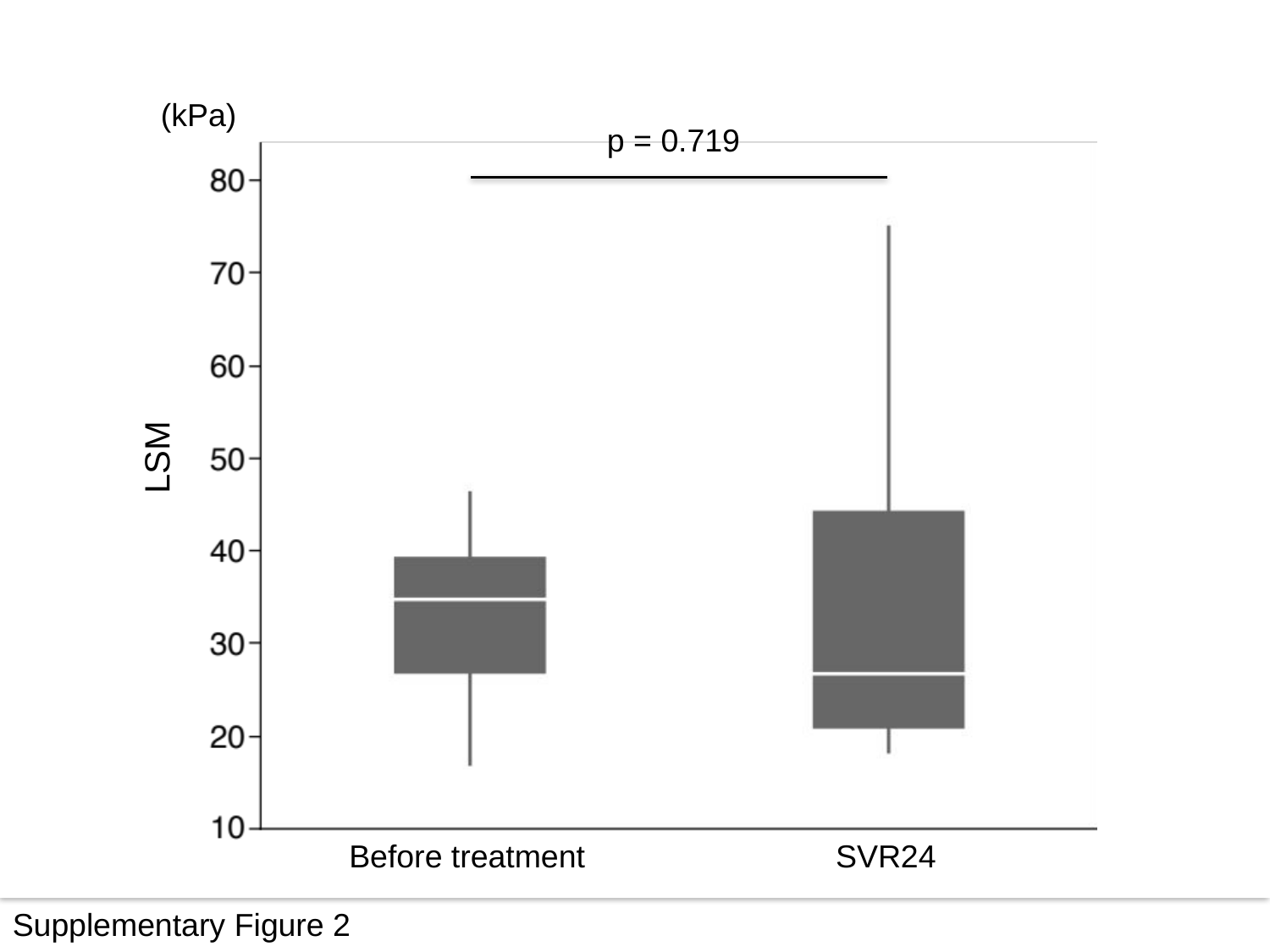

(kPa)
 p = 0.719
LSM
Before treatment
SVR24
Supplementary Figure 2

Supplement: Supplementary file 2 — Supplementary file2 Liver stiffness measurement (LSM) was 35 (27–39) kPa before sofosbuvir/velpatasvir treatment and decreased to 27 (21–44) kPa after sustained virologic response at 24 weeks post-treatment (SVR24). This decrease was not statistically significant (p=0.719) (PPTX 73 KB) [file 535_2023_1963_MOESM2_ESM.pptx]

## Slide 1
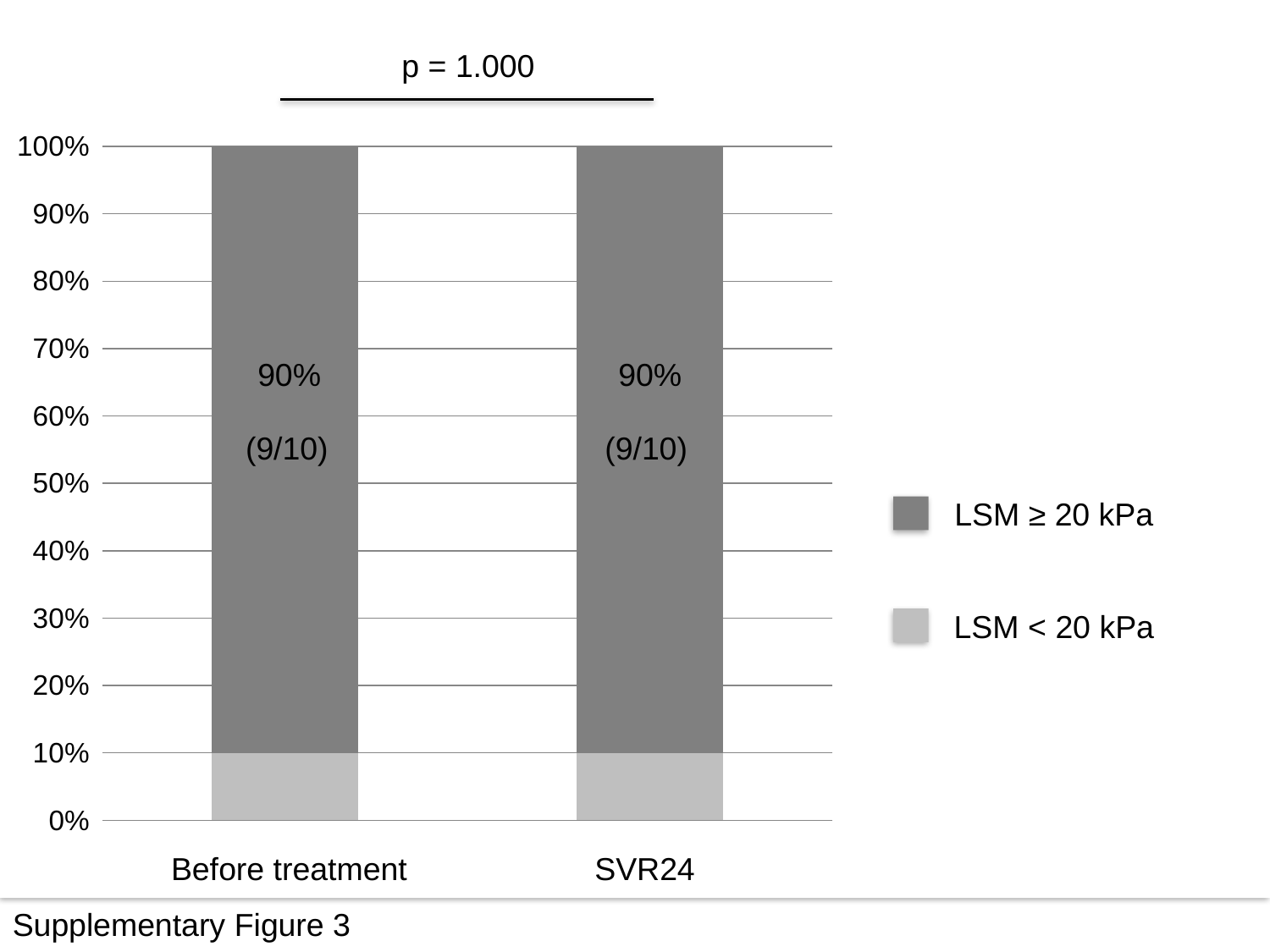

p = 1.000
### Chart
| Category | | |
|---|---|---|90%
90%
(9/10)
(9/10)
LSM ≥ 20 kPa
LSM < 20 kPa
Before treatment
SVR24
Supplementary Figure 3

Supplement: Supplementary file 3 — Supplementary file3 Percentage of patients with a liver stiffness measurement (LSM) ≥ 20 kPa was 90% (9/10) before sofosbuvir/velpatasvir treatment and remained at 90% (9/10) after sustained virologic response at 24 weeks post-treatment (SVR24) (p=1.000) (PPTX 60 KB) [file 535_2023_1963_MOESM3_ESM.pptx]

## Slide 1
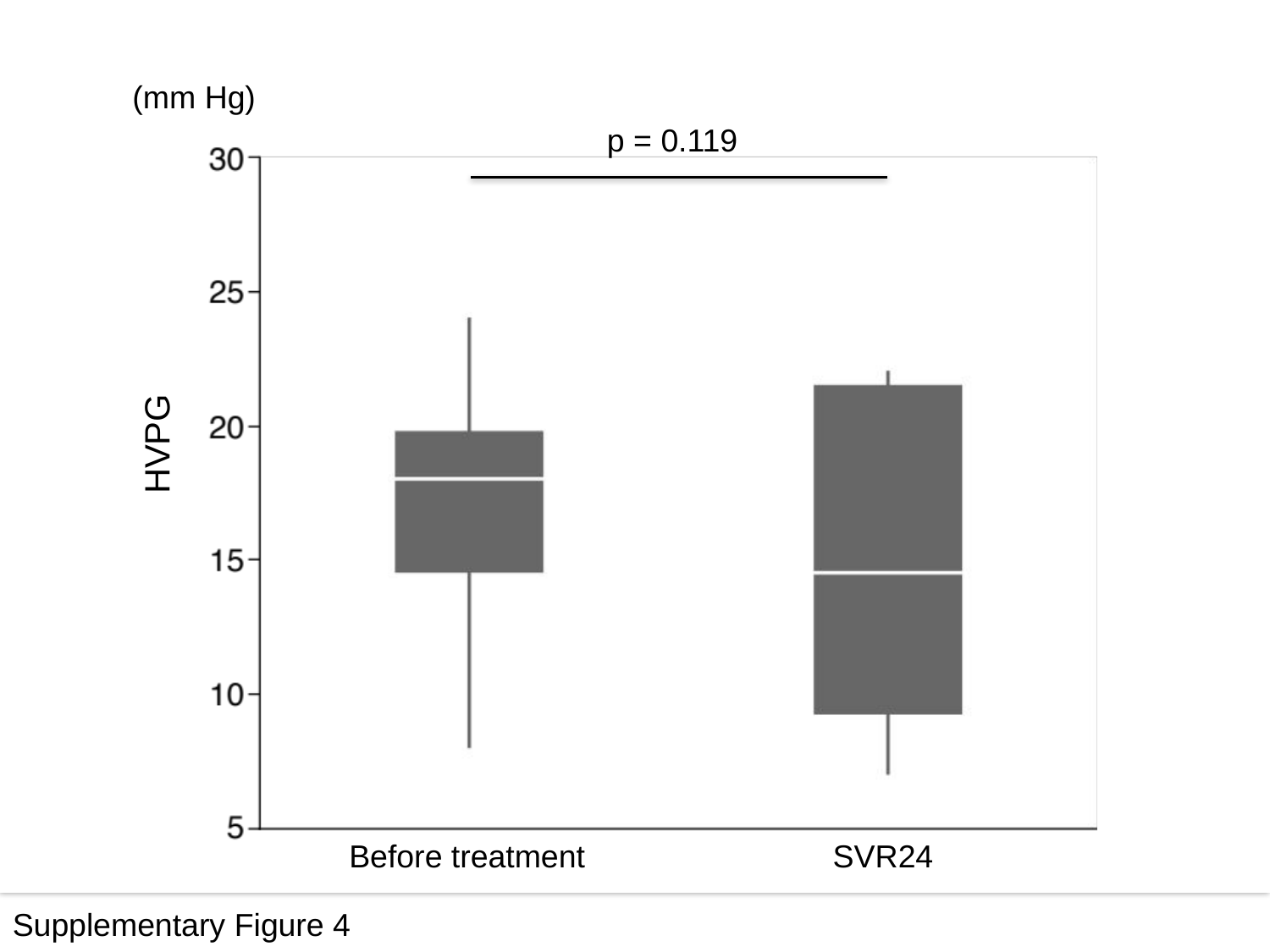

(mm Hg)
 p = 0.119
HVPG
Before treatment
SVR24
Supplementary Figure 4

Supplement: Supplementary file 4 — Supplementary file4 Hepatic venous pressure gradient (HVPG) was 18 (15–20) mmHg before sofosbuvir/velpatasvir treatment and decreased to 15 (9–22) mmHg after sustained virologic response at 24 weeks post-treatment (SVR24). However, this decrease was not statistically significant (p=0.119) (PPTX 76 KB) [file 535_2023_1963_MOESM4_ESM.pptx]

## Slide 1
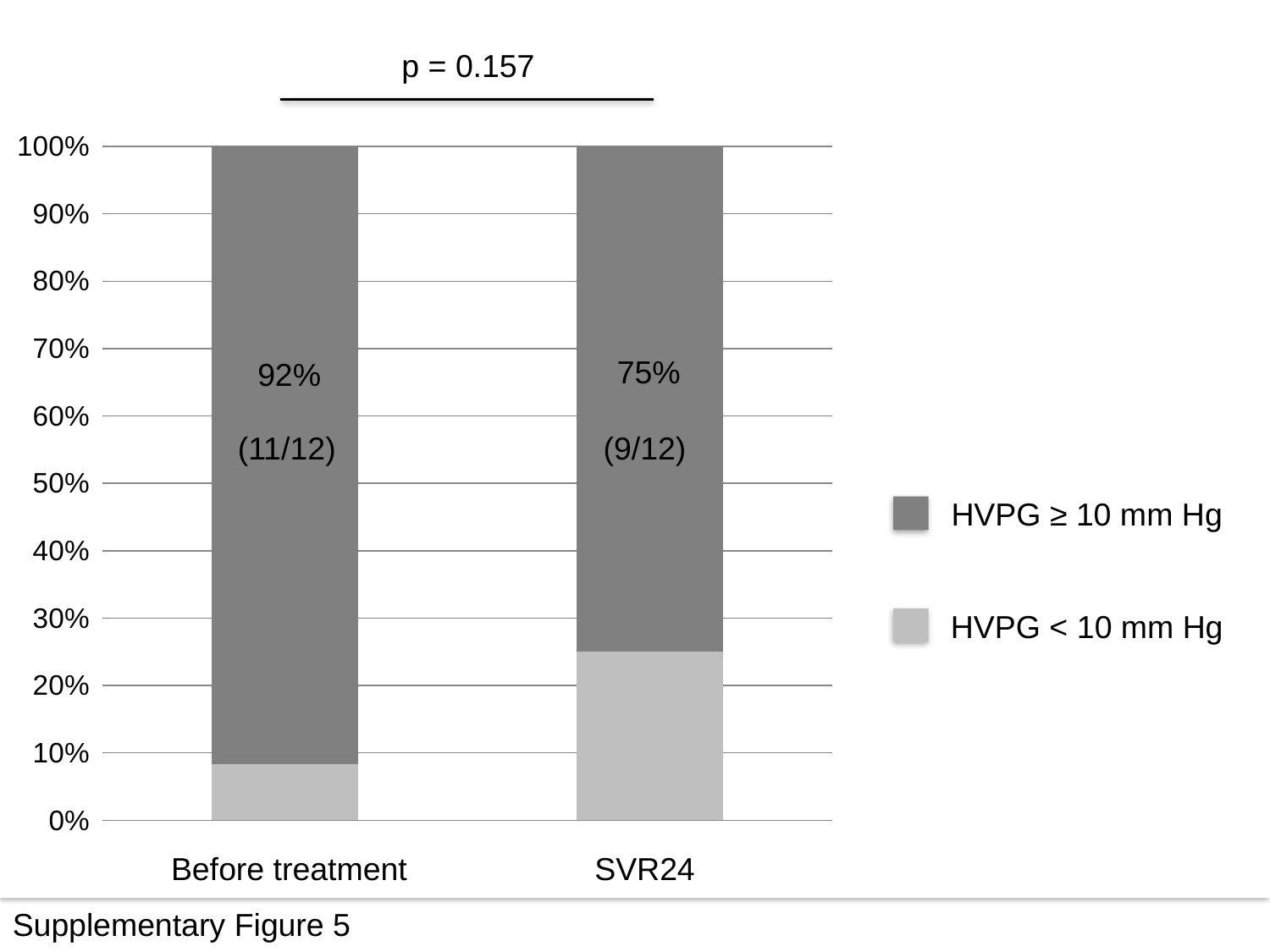

p = 0.157
### Chart
| Category | | |
|---|---|---|75%
92%
(11/12)
(9/12)
HVPG ≥ 10 mm Hg
HVPG < 10 mm Hg
Before treatment
SVR24
Supplementary Figure 5

Supplement: Supplementary file 5 — Supplementary file5 Percentage of patients with clinically significant portal hypertension (CSPH), defined as a hepatic venous pressure gradient (HVPG) ≥ 10 mmHg, was 92% (11/12) before sofosbuvir/velpatasvir treatment and decreased to 75% (9/12) after sustained virologic response at 24 weeks post-treatment (SVR24). However, the decrease was not statistically significant (p=0.157) (PPTX 60 KB) [file 535_2023_1963_MOESM5_ESM.pptx]
