# Supplementary material for: Short-term hepatocyte function and portal hypertension outcomes of sofosbuvir/velpatasvir for decompensated hepatitis C-related cirrhosis
Source: J Gastroenterol. 2023 Feb 2;58(4):394–404. doi: 10.1007/s00535-023-01963-2 (PMC10049944; doi:10.1007/s00535-023-01963-2)
Supplement: Supplementary file 6 — Supplementary file6 (DOCX 27 KB) [file 535_2023_1963_MOESM6_ESM.docx]

**Supplementary Table 1.** Baseline characteristics of the decreased LSM group and increased LSM group (*n* = 10)

| Characteristic | Decreased LSM (*n* = 6) | Increased LSM (*n* = 4) | p |
| --- | --- | --- | --- |
| CPT score | 7 (7–9) | 8 (7–9) | 0.718 |
| ALBI score | -1.34 (-1.06 to -1.93) | -1.46 (-1.19 to -2.15) | 0.670 |
| MELD score | 10 (9–12) | 11 (9–13) | 0.664 |
| Type 4 collagen 7S (ng/mL) | 15.4 (9.8–16.6) | 13.8 (8.5–16.4) | 0.522 |
| M2BPGi (C.O.I) | 14.5 (9.5–18.6) | 13.3 (9.6–15.6) | 0.670 |
| FIB-4 index | 8.23 (5.19–12.48) | 7.14 (6.13–10.55) | 1.000 |
| PSS diameter (mm) | 8 (7–11) | 6 (5–10) | 0.194 |
| Liver volume (mL) | 1077 (982–1283) | 838 (809–1025) | 0.053 |
| Spleen volume (mL) | 311 (226–877) | 166 (98–516) | 0.180 |
| L/S ratio | 3.4 (1.5–4.8) | 6.2 (1.6–8.3) | 0.297 |
| LHL 15 | 0.65 (0.58–0.75) | 0.77 (0.54–0.78) | 0.796 |
| HH15 | 0.84 (0.73–0.87) | 0.79 (0.75–0.88) | 0.796 |
| LSM (kPa) | 35 (32–39) | 32 (19–42) | 0.669 |
| HVPG (mm Hg) | 19 (16–21) | 17 (14–18) | 0.238 |

LSM, liver stiffness measurement; CPT, Child-Pugh-Turcotte; ALBI, albumin-bilirubin; MELD, model for end-stage liver disease; M2BPGi, mac-2 binding protein glycosylation isomer; PSS, portosystemic shunt; L/S ratio, liver-to-spleen volume ratio; LHL15, hepatocyte receptor index; HH15, blood clearance index; HVPG, hepatic venous pressure gradient.

Data are shown as median (interquartile range) and were compared using the Mann-Whitney U test.
